# EHMTI-0235. Hemodialysis-related headache

**DOI:** 10.1186/1129-2377-15-S1-C58

**Published:** 2014-09-18

**Authors:** D Uludüz, B Göksan, G Gözübatik Çelik, N Akkaya, M Sohtaoglu Sevindik, U Uygunoglu, S Saip, F Kircelli, A Sezen, A Siva

**Affiliations:** 1 Neurology Department, Istanbul UNiversity Cerrahpasa Medical Faculty, Istanbul, Turkey; 2 Cerrahpasa Medical Faculty, Istanbul UNiversity, Istanbul, Turkey; 3 Department of Internal Medicine Nephrology Division, Istanbul University Cerrahpasa Medical Faculty, Istanbul, Turkey

## Background

Hemodialysis (HD) increases expected lifetime of the patients minimizing the effects of neurological complications. However, dialysis may in itself, also have prejudicial effects on the nervous system. Headache (HDH) is the most common reported complaint among HD patients.

## Aim

In the presented prospective study we aimed to further understand the HDH frequency and clinical characteristics in a large group of patients undergoing HD under standard dialysis programme with bicarbonate dialysate.

## Method

A total of 494 patients were included. Patients undergoing HD sessions with no complaints of headache or any other neurological disorders were selected as control group. For HDH and control subjects arterial systolic and diastolic blood pressure, body weight, serum levels of BUN and creatinine were measured before and after one HD session.

## Results

175 patients (35.42%) were diagnosed with HDH and 275 patients were selected as controls. Headache was started with a mean of 2.90±0.72 hours of the hemodialysis session. The pain was usually throbbing in bi-frontal region , the mean duration of headache was 6.1±7.7h, and VAS score was 5.63±2.02.. The difference between pre- and post- dialysis values of BUN in patients with HDH was statistically significant,. Patients with HDH showed significantly higher mean systolic and diastolic blood pressure pre-dialysis values comparing with control group, whereas post-dialysis values did not differ between the two groups.

## Conclusion

HDH may cause disability to the patient and changing dialysis parameters or methods can prevent the attacks and disability.

No conflict of interest.

